# Anxiety type modulates immediate versus delayed engagement of attention-related brain regions

**DOI:** 10.1002/brb3.157

**Published:** 2013-08-01

**Authors:** Jeffrey M Spielberg, Angeline A De Leon, Keith Bredemeier, Wendy Heller, Anna S Engels, Stacie L Warren, Laura D Crocker, Bradley P Sutton, Gregory A Miller

**Affiliations:** 1 University of Illinois at Urbana-ChampaignChampaign, Illinois; 2 University of CaliforniaBerkeley, California; 3 The Ohio State UniversityColumbus, Ohio; 4 Pennsylvania State UniversityUniversity Park, Pennsylvania; 5 University of DelawareNewark, Delaware; 6 University of KonstanzKonstanz, Germany

**Keywords:** Anxiety, anxious apprehension, anxious arousal, attention, fMRI, habituation, negative valence

## Abstract

**Background** Habituation of the fear response, critical for the treatment of anxiety, is inconsistently observed during exposure to threatening stimuli. One potential explanation for this inconsistency is differential attentional engagement with negatively valenced stimuli as a function of anxiety type. **Methods** The present study tested this hypothesis by examining patterns of neural habituation associated with anxious arousal, characterized by panic symptoms and immediate engagement with negatively valenced stimuli, versus anxious apprehension, characterized by engagement in worry to distract from negatively valenced stimuli. **Results** As predicted, the two anxiety types evidenced distinct patterns of attentional engagement. Anxious arousal was associated with immediate activation in attention-related brain regions that habituated over time, whereas anxious apprehension was associated with delayed activation in attention-related brain regions that occurred only after habituation in a worry-related brain region. **Conclusions** Results further elucidate mechanisms involved in attention to negatively valenced stimuli and indicate that anxiety is a heterogeneous construct with regard to attention to such stimuli.

## Introduction

Excessive anxiety is associated with hyperactivity in fear systems (Lang et al. 2006). In response to cues associated with threat, a fear response is triggered, which can become debilitating and uncontrollable. Although vigilance for danger cues can be useful in threatening situations, exaggerated selectivity for negatively valenced information may be more distracting and intrusive than useful in nonthreatening situations, as appears to be the case in anxiety disorders (Mathews and MacLeod 1985).

Seemingly paradoxically, exposure to threat-related stimuli can lead to habituation of the fear response by gradually diminishing the potency of feared stimuli (Head and Gross 2003). However, some studies have failed to find habituation during exposure to negatively valenced stimuli (Smith et al. 2005; Wendt et al. 2008). Failure to habituate may be due to recruitment of cognitive avoidance strategies, which direct attention away from the threatening aspects of stimuli (Williams et al. 1988). Thus, the process of habituation seems to vary as a function of attentional engagement: directing attention toward negatively valenced stimuli results in habituation, whereas engaging in distraction does not (Telch et al. 2004).

Borkovec and colleagues proposed that worrying provides distraction from the full-blown intensity of fear experienced as a result of engagement in catastrophic, threat-related imagery (Borkovec et al. 2004), and research supports the association between worry and failure to habituate (Borkovec and Inz 1990; Thayer et al. 2000). Therefore, worry may serve as a negatively reinforced coping mechanism, because it results in avoidance of a full fear response by redirecting attention, thus maintaining the fear response over time. Inconsistencies in findings regarding habituation may therefore be due to individual differences in the tendency to employ cognitive avoidance strategies (e.g., worry).

These two responses to negatively valenced stimuli (engagement in worry vs. an immediate fear response) are thought to reflect two distinct types of anxiety (Nitschke et al. 1999). Anxious apprehension, characterized by worry, is the cardinal feature of generalized anxiety disorder (GAD; Barlow 1986). Anxious arousal, characterized by sympathetic hyperarousal and somatic tension, is an important component of panic disorder. Increased neural activation to negative words in Broca's area has been associated with anxious apprehension (Engels et al. 2007, 2010), consistent with the role of this area in verbal processes (Zatorre et al. 1996). Increased activation to negative words in right inferior and middle temporal gyri (ITG, MTG) has been associated with anxious arousal (Engels et al. 2007), consistent with the role of this area in the identification of salient and unexpected stimuli (Compton et al. 2003; Corbetta et al. 2008) and theorizing regarding the role of this area in threat response (for review, see Nitschke et al. 2000).

To date, research has not compared neural patterns of habituation to negatively valenced stimuli in anxious apprehension and anxious arousal. Differences in patterns of habituation associated with these two types of anxiety may explain inconsistencies observed in the literature. The present study explored this hypothesis by contrasting the time courses of responding to negatively valenced stimuli associated with anxious apprehension and anxious arousal. The present study examined whether responses to negatively valenced stimuli differed between the first and second halves (each approximately 6 min long) of an emotion-word Stroop task (Williams et al. 1996; see Buhle et al. 2010; for a discussion of the processes thought to be engaged in this task). In this task, participants report the ink color of emotionally valenced words while trying to ignore their meaning. Evidence suggests that reading negatively valenced words can induce preparatory responses to threat (e.g., potentiated startle responses, Herbert and Kissler 2010). Thus, negatively valenced words can be difficult to ignore due to attentional engagement with threat stimuli. Accordingly, anxiety has been associated with slowed color naming for negatively valenced words (Bar-Haim et al. 2007). An emotion-word Stroop task was employed in the present study, because neural habituation has been observed in an emotion-word Stroop task (Canli et al. 2004), and the emotion-word Stroop has been shown to elicit individual differences in processing of negatively valenced stimuli (Koven et al. 2003).

Several brain areas were examined, including areas expected to show specific associations with each anxiety type, as well as areas generally associated with the direction of attention toward negatively valenced stimuli. Given that worry is a subset of verbal rehearsal (Borkovec and Inz 1990) and that Broca's area is consistently associated with verbal rehearsal (Zatorre et al. 1996), it was hypothesized that engagement in worry would be reflected in Broca's area activation. Therefore, Broca's area was expected to show a specific association with anxious apprehension. Anxious arousal was expected to show a specific association with right MTG/ITG, given evidence indicating that individuals high in anxious arousal show hyperactivation in this region (Engels et al. 2007).

Superior prefrontal cortex, including dorsolateral prefrontal cortex (DLPFC) and frontal eye field (FEF), was examined for association with both anxiety types. DLPFC and FEF have been associated with both top-down maintenance of attention and reorientation of attention toward unexpected, salient stimuli (Corbetta et al. 2008). Right DLPFC has also been related to avoidance motivation and related constructs (Shackman et al. 2009; Spielberg et al. 2011b, 2012), which are thought to organize goal pursuit related to undesirable outcomes (Elliot 1999) and are positively correlated with both anxiety types (Spielberg et al. 2011a). Additionally, research suggests that, when the potential for threat hinders goal-directed processing in right DLPFC, concurrent goal-directed behavior is disrupted (Shackman et al. 2011a). In contrast, left DLPFC may be specifically involved in pleasant emotional valence/approach motivation (e.g., Herrington et al. 2005; Spielberg et al. 2011b) and appears to exhibit differential habituation to pleasant/approach-related stimuli (relative to threat stimuli, Wright et al. 2001). Thus, it is likely that right rather than left DLPFC will exhibit anxiety-dependent habituation to negatively valenced stimuli.

Anterior cingulate cortex (ACC) and amygdala were also examined for association with both anxiety types. Evidence suggests that ACC is involved in diverting attentional resources toward negatively valenced stimuli (Devinsky et al. 1995), and activation in this region habituates to repeated presentations of emotional stimuli (Phan et al. 2003). Amygdala has been strongly implicated in responding to negatively valenced stimuli (Phelps 2009) and habituates to repeated presentations of such stimuli (Breiter et al. 1996). Although a number of other brain regions are thought to be involved in the general direction of attention (e.g., intraparietal sulcus), the present study focused on this subset of regions because of the evidence that they are involved in the direction of attention specifically to negatively valenced stimuli.

Table 1 summarizes the main hypotheses. Given the characteristic vigilance associated with anxious arousal, it was hypothesized to be associated with a strong response to negative words during the first half of the task in attention-related brain regions (i.e., right MTG/ITG, DLPFC, and FEF). This initial response was hypothesized to habituate by the second half of the task. Additionally, this temporal pattern was hypothesized to be reflected in overt behavioral performance.

**Table 1:** Summary of hypotheses

	Primary habituation analyses			PPI analyses
		
	Broca's area	R MTG/ITG	Attention-related brain regions	Connectivity between Broca'sarea and attention regions
Anxious apprehension	**↓**over time	n/a	**↑**over time	**↓**connectivity
Anxious arousal	n/a	**↓**over time	**↓**over time	–

R, right; MTG, middle temporal gyrus; ITG, inferior temporal gyrus. Attention-related brain regions, dorsolateral prefrontal cortex, frontal eye fields, anterior cingulate, and amygdala. **↓**over time, anxiety type predicted to show greater decrease in activation over time; **↑**over time, anxiety type predicted to show greater increase in activation over time; **↓**connectivity, anxiety type expected to show a greater decrease in condition-dependent connectivity; –, no prediction made.

In contrast, anxious apprehension was not expected to be associated with an increased initial fear response, given that worry can prevent full engagement with negative words. Rather, anxious apprehension was predicted to be associated with engagement in worry in the first half of the task. The full time course of anxious apprehension is difficult to predict, given the dearth of available research in this area. However, based on research suggesting that worry is cognitively taxing (Hayes et al. 2008), the most likely pattern appears to be that worry decreases over time, as the resources engaged by worry become depleted. If worry decreases, attention to negative words should increase (because cognitive avoidance is no longer occurring).

Anxious apprehension was hypothesized to show greater activation in Broca's area in the first half of the task, which would habituate by the second half. Additionally, as activation in Broca's area habituates, activation in attention-related brain regions (i.e., DLPFC, FEF, ACC, and amygdala) should increase (i.e., greater activation in the second half of the task than in the first). This hypothesis was tested directly using psychophysiological interaction analysis (PPI) of the time series of Broca's area and attention-related regions. Activity in Broca's area was expected to show a greater negative correlation with activity in attention-related regions during the negative condition than during the neutral condition for individuals higher in anxious apprehension.

With regard to the relationship between anxious apprehension and overt behavior, it is possible that both worry and attentional engagement with stimuli will interfere with performance. If so, and if the hypotheses above are supported (i.e., habituation in worry, increase in attention over time), anxious apprehension would not be associated with habituation in behavior, because a behaviorally interfering process is occurring at all times (i.e., worry in the first task half, attentional engagement with stimuli in the second). In order to test whether the effects of worry and attentional engagement on behavior cancel out, mediation analyses were carried out, with habituation of behavior as the dependent variables, anxious apprehension as the independent variable, and habituation of activation in Broca's area and attention-related regions as mediators.

In summary, we anticipated that the two anxiety types would be characterized by different patterns of what has been termed affective chronometry (Davidson 1998). Specifically, we hypothesized that anxious arousal would be characterized by a relatively quick rise time to peak attentional engagement with negatively valenced words, along with a relatively rapid recovery to baseline. In contrast, we expected that anxious apprehension would be characterized by a relatively slow rise to peak attentional engagement and, potentially, a slower recovery time.

## Methods

### Participants and questionnaires

Participants were 104 right-handed, native English-speaking undergraduates with normal color vision.^1^ Participants were recruited from a larger pool of undergraduates (*n* = 2723) based on three scales: the Penn State Worry Questionnaire (PSWQ, Meyer et al. 1990), the Anxious Arousal scale of the Mood and Anxiety Symptom Questionnaire (MASQ-AA, Watson et al. 1995a), and the Loss of Interest subscale of the Anhedonic Depression scale of the Mood and Anxiety Symptom Questionnaire (MASQ-AD-LI). Specifically, participants were contacted if (1) they scored at or above the 80th percentile (PSWQ ≥ 63, MASQ-AA ≥ 33, MASQ-AD-LI ≥ 22) on one of the three psychopathology dimensions and at or below the 50th percentile (PSWQ ≤ 49, MASQ-AA ≤ 25, MASQ-AD-LI ≤ 17) on the other two dimensions, (2) they scored at or above the 80th percentile on all three psychopathology dimensions, or (3) they scored at or below the 50th percentile on all three psychopathology dimensions. The present investigation utilized a dimensional analytic approach because this approach is often associated with greater power (Preacher et al. 2005).

Participants' data were excluded if they had head motion greater than 2 mm relative to the previous volume or 3.3 mm relative to the middle time series volume (*n* = 5), functional magnetic resonance imaging (fMRI) data exhibiting visible, stimulus-correlated, motion-related artifact (*n* = 15), incomplete questionnaire data (*n* = 1), or mean reaction time (RT; *n* = 3), number of errors (*n* = 4), or questionnaire data (*n* = 1) greater than 3 standard deviations (SDs) from the mean. This left 75 participants (56% female, mean age 19.1 years, SD 1.02 years) with usable data.

After recruitment, participants completed the PSWQ and MASQ a second time. In order to best capture the level of anxiety at the time that the fMRI data were collected, only the data from the second administration of the questionnaires were used in fMRI analyses (all relationships remained significant when using the average value of the two administrations). For PSWQ, a 16-item measure used to assess anxious apprehension, participants rated how characteristic (1 = “not at all”, 5 = “very typical”) each item was of them. Participants completed the MASQ 17-item Anxious Arousal scale and the 8-item Loss of Interest Anhedonic Depression subscale (Nitschke et al. 2001; Bredemeier et al. 2010).^2^ For both MASQ scales, participants rated how much they experienced each item during the previous week (1 = “not at all”, 5 = “extremely”).

### Stimuli and experimental design

Participants completed two tasks, an emotion-word and a color-word Stroop (duration of each task = 12 min 20 sec) in fMRI and electroencephalography (EEG) sessions. Findings from color-word Stroop are not presented here, beyond minor analyses to assess specificity, and findings from EEG sessions are not presented here (for detailed analyses of EEG data, see Sass et al. 2010 and Silton et al. 2010; and of color-word data, see Spielberg et al. 2011b). Order of presentation of tasks and sessions was counterbalanced across participants.^3^

The emotion-word Stroop task consisted of blocks of positive, neutral, and negative words. Findings from the positive word blocks are not presented here, beyond minor analyses to assess specificity (for detailed analyses of these data see Spielberg et al. 2012; and Warren et al. 2010). In each trial, a word was presented in one of four possible ink colors (red, yellow, green, blue), and participants were instructed to press one of four buttons to indicate the color of the ink in which the word appeared. Word meaning was irrelevant to performance of the task. Descriptive statistics for the stimuli are presented in Table 2. Each word was presented for 1500 msec, followed by a fixation cross presented for an average of 500 msec, with a variable inter-trial interval (2000 ± 225 msec) between trial onsets. Word presentation and reaction-time measurement were controlled by STIM software (James Long Company, Caroga Lake, NY).

**Table 2:** Stimulus characteristics

	Pleasant words	Unpleasant words	Neutral words
Average valence	7.8	2.5	5.2
Average arousal	6.6	6.5	3.8
Average frequency	52.4	60.0	60.0
Average word length	5.8	5.4	5.3

Word stimuli were selected from the Affective Norms for English Words (ANEW) set (Bradley and Lang 1999). Valence and arousal data from the ANEW set are represented on a scale ranging from 1 to 9, with 9 representing the most pleasant and most arousing ratings, respectively. Frequency information was obtained from Toglia and Battig (1978).

The task involved 16 word blocks (four positive, four negative, and eight neutral blocks), with 16 trials in each block.^4^ Blocks were presented in one of four orders, counterbalanced across participants. Each order consisted of four “super-blocks” containing four word blocks each (two emotion, two neutral), and between every “super-block” participants either viewed a fixation cross or were told to rest. Each super-block contained the same order of word blocks, and emotion and neutral blocks always alternated within super-blocks. Counterbalancing orders varied whether an emotion or neutral block was presented first within the super-blocks (e.g., neg, neu, pos, neu vs. neu, neg, neu, pos) and whether the negative condition was presented before the positive condition across super-blocks (e.g., neg, neu, pos, neu vs. pos, neu, neg, neu). In summary, each half of the task contained two super-blocks, and the halves contained an equal number of word blocks per condition (two negative, two positive, four neutral blocks). Thus, the first and second halves of the task were identical in form, although the actual words used as stimuli differed in each block (no words were repeated).

### Behavioral analysis

Behavioral data were analyzed by computing average RT for correct trials and number of errors for each participant in the negative and neutral conditions separately for each half of the task. A Time (first half of the task vs. second half of the task) × Emotion (negative words vs. neutral words) repeated-measures general linear model (GLM) was conducted (using SPSS v19), with PSWQ, MASQ-AA, and MASQ-AD-LI entered as continuous predictors. The effects of particular interest were the Time × Emotion × PSWQ and Time × Emotion ×MASQ-AA interactions, which tested whether habituation in the response to negative words was moderated by the anxiety types.

### fMRI data collection

MRI data were collected using a 3T Siemens Allegra (Siemens Medical Solutions USA, Inc., Malvern, PA). The fMRI data were 370 three-dimensional images acquired using a Siemens gradient-echo echo-planar imaging sequence (TR 2000 msec, TE 25 msec, flip angle 80°, FOV = 220 mm). Each image consisted of 38 oblique axial slices (slice thickness 3 mm, 0.3 mm gap, in-plane resolution 3.4375 mm by 3.4375 mm). After the fMRI acquisition, a 160-slice MPRAGE structural sequence was acquired (spatial resolution 1 mm, isometric), which was used to warp the participant's functional data into standard space.

### fMRI data reduction and preprocessing

FEAT (FMRI Expert Analysis Tool, http://www.fmrib.ox.ac.uk/analysis/research/feat/), part of the FSL (FMRIB Software Library, http://www.fmrib.ox.ac.uk/fsl) analysis package, was used to process each participant's functional brain images and carry out group analyses. A high-pass filter was used to remove drift in MRI signal intensity, and functional data were motion-corrected and spatially smoothed using a 5 mm (full-width half-max) 3D Gaussian kernel. Temporal low-pass filtering was carried out using AFNI's 3dDespike tool (http://afni.nimh.nih.gov/), followed by fieldmap correction.

### fMRI data processing

#### Within-participant analyses

Regression analyses were performed on the processed functional time series using FILM (FMRIB's Improved Linear Model with autocorrelation correction, Woolrich et al. 2001). Two predictors were created for each condition (positive, neutral, and negative). In order to split the data into halves, the first two blocks of each emotion condition were modeled in one predictor and the second two blocks in a separate predictor. Given that there were eight neutral blocks (rather than four), the first four neutral blocks were modeled separately from the second four neutral blocks. The functional data were not actually split in half and halves analyzed separately; rather, the entire time course of the data was analyzed simultaneously, and the halves were modeled by separate predictors.

Predictors modeled entire blocks of words, rather than individual trials, because the timing of the task (TR = 2 sec and ITI = ∼2 sec), did not allow for differentiation between block-level and trial-specific variance. An additional predictor was created to model the rest condition (fixation was left un-modeled). Predictors were convolved with a canonical hemodynamic response and entered into a GLM. Each predictor yielded a per-voxel effect-size parameter estimate (*β*) map representing the magnitude of activation associated with that predictor. Contrasts of *β* values were created to quantify the level of habituation in the response to negative words across time. Specifically, for the first and second halves of the task individually, the *β* for neutral was subtracted from the *β* for negative, isolating the effect due to the negative valence and high arousal of the words in that half. Next, the two halves of the task were contrasted by subtracting *β*'s for the first half from *β*'s for the second half, isolating the change in effect for negative words over time. Negative values of this contrast represent decreased response to negative words over time (habituation), whereas positive values of this contrast represent increased response to negative words over time. Single-subject *β* maps were warped into a common stereotaxic space (ICBM152 2009a Nonlinear Symmetric, 1 mm × 1 mm ×1 mm T1 Atlas, Fonov et al. 2009) using FNIRT (FMRIB's Non-Linear Image Registration Tool, Andersson et al. 2007) for use in group-level analyses.

#### Group-level analyses

Hierarchical linear modeling using FLAME (FMRIB's Local Analysis of Mixed Effects; Woolrich et al. 2001) was carried out to determine the simple effect of habituation to negative stimuli and whether anxious apprehension and anxious arousal moderated habituation. To determine the simple effect of habituation, a mixed-effects *t*-test of the mean value of the single-subject level contrast was conducted for each voxel. To examine moderation, the contrast values for each participant were entered as a dependent variable in a mixed-effects regression with PSWQ, MASQ-AA, and MASQ-AD-LI entered as continuous predictors. MASQ-AD-LI was entered as a covariate in order to remove variance associated with general distress that is common to depression and anxiety (Clark and Watson 1991), given the present interest in the unique variance associated with anxiety.^5^ Rerunning the analyses without MASQ-AD-LI as a predictor revealed virtually identical findings (see Miller and Chapman 2001, on this use of analysis of covariance (ANCOVA), indicating that inclusion of MASQ-AD-LI did not bias the findings. Additionally, rerunning analyses with each anxiety type as the sole predictor revealed virtually identical findings, indicating that the findings were not an artifact of removing shared variance.

For each voxel, for each predictor of interest (PSWQ and MASQ-AA), a *t-*test was carried out on the *β* values to identify voxels in which there was significant moderation. *T*-tests were one-tailed in the direction of the a priori prediction (i.e., negative for anxious apprehension and Broca's area, positive for all other comparisons). In order to correct for multiple comparisons, AFNI's AlphaSim (Ward 2000) was used to obtain cluster-size thresholds, which, in combination with an individual voxel-level threshold, ensured that each cluster was significant at *P* ≤ 0.05. Contiguous voxels within a cluster were defined as those voxels that were connected by a face or an edge (not merely by a corner).

Small volume correction was used for a priori regions of interest, using masks from the Harvard-Oxford probabilistic atlas available with FSL. A mask of left inferior frontal gyrus (IFG; including Broca's area)^6^ was created for the one-tailed (negative direction) *t*-test of the relationship between brain activation and PSWQ (cluster-size threshold = 390 mm^3^). For the one-tailed (positive direction) *t*-test of the relationship between PSWQ and the other brain regions, the mask included bilateral superior prefrontal cortex, ACC, and amygdala (cluster-size threshold = 819 mm^3^).^7^ For the one-tailed (positive direction) *t*-test of the relationship between brain activation and MASQ-AA, the mask included right MTG/ITG, bilateral superior prefrontal cortex, ACC, and amygdala (cluster-size threshold = 819 mm^3^). For each mask, an individual voxel-level threshold of *P* = 0.03 was used, and a cluster-size threshold was computed and used only for voxels within the mask. In areas where PSWQ and MASQ-AA exhibited effects in opposing directions, direct comparisons were computed to test whether the effects differed significantly. These comparisons (tests of the difference of dependent *β*s) were computed in a voxel-wise manner and thresholded as described above.

In order to determine whether observed effects were driven by changes in negative, neutral, or both stimuli, average *β* values for each cluster for each participant were extracted separately for negative and neutral (relative to fixation) for each half. This provided four values for each cluster, for each participant (negative first half, negative second half, neutral first half, and neutral second half). Habituation variables were then created separately for negative and neutral by subtracting the average *β* for the first half from the average *β* from the second half. Partial correlations with the relevant anxiety type (PSWQ or MASQ-AA) were computed, with the variance associated with the other anxiety type and MASQ-AD-LI partialled out.

#### Psychophysiological interaction analyses

PPI analyses were performed on the preprocessed functional time series for each participant using FILM and did not differentiate between the first and second half of the time series. A cluster in Broca's area, identified during the main analyses, was used as the seed cluster. For each participant, the cluster was warped from Montreal Neurological Institute (MNI) space to functional space, and the time series was extracted. Six predictors were entered in the within-participant analyses: (1) the time series of Broca's area, (2) a predictor that modeled the difference between negative and neutral conditions (coded as 1 during the negative condition, −1 during the neutral condition, 0 at all other times), (3) the interaction of these two predictors, and (4–6) three predictors of no interest that modeled the variance associated with the positive condition, the rest condition, and the sum of the negative and neutral conditions. The negative versus neutral predictor and the three predictors of no interest were convolved with a gamma function to better approximate the temporal course of the BOLD hemodynamic response function (this convolution was performed on the negative vs. neutral predictor prior to creating the interaction term).

Group inferential statistical analyses were carried out using FLAME. The *β* maps corresponding to the PPI interaction term were regressed on the psychopathology questionnaires. Thresholding and correction for multiple comparisons were conducted in the manner described above. One-tailed *t*-tests were used to test the PSWQ *β* map in the a priori regions of interest. Two-tailed *t*-tests were used in conjunction with a whole-brain gray-matter mask to examine the *β* maps for MASQ-AA and MASQ-AD-LI, because no a priori hypotheses about these predictors were made.

To determine whether the observed effects held separately in the first and second halves of the task, time series data for each cluster were extracted from the negative and neutral blocks, and the Broca's area time course X PSWQ X Emotion interaction was tested separately for each half of the task in a hierarchical linear model using the Mixed procedure in SPSS v19. Participant was the nesting variable, block and timepoint were repeated factors, and a lag 1 autoregressive function was used.

#### Lateralization analyses

Lateralization was tested using a locally written Matlab program that conducted a repeated-measures homogeneity of slopes GLM analysis, with hemisphere as the repeated measure, PSWQ, MASQ-AA, and MASQ-AD-LI scores as continuous predictors, and fMRI activation for the single-subject level contrast as the dependent variable. This ANCOVA was conducted on a per-voxel basis, and the resultant *β* maps were thresholded in the manner described above, with the exception that *F*-tests were used.

Because testing laterality determines whether the *β* in a voxel in the right hemisphere is significantly different from the *β* in the homologous voxel in the left hemisphere, half as many tests were conducted as in a nonlaterality analysis. Therefore, a mask containing only the right-hemisphere portion of the superior prefrontal mask was used.

#### Anxious apprehension ROI mediation analyses

Mediation analyses were carried out in SPSS v19 using the INDIRECT macro (Preacher and Hayes 2008). PSWQ was entered as an independent variable, with MASQ-AA and MASQ-AD-LI entered as covariates. To isolate behavioral habituation to negative stimuli, composite RT and error variables were created. Specifically, interference due to negative words (i.e., negative – neutral) in the first half of the task was subtracted from interference due to negative words in the second half of the task. Mediators were average habituation in activation to negative words in each ROI associated with PSWQ.

### Tests of specificity to negative stimuli

#### Examination of positive stimuli

In order to ensure that present findings were driven by the negative valence of the stimuli rather than their arousal value, the relationship between PSWQ/MASQ-AA and habituation of activation related to positive words was examined in two ways. First, the analyses above were rerun with the exception that the single-subject contrast was positive minus neutral (as opposed to negative minus neutral) and that 2-tailed tests were used. Apart from this difference, these analyses were identical to the main analyses.

Second, for each ROI identified in the main (i.e., negative minus neutral) analyses, the average *β* (across voxels) was computed for positive and neutral (vs. baseline), for each half of the session, for each participant. These values were entered into a repeated-measures GLM (using SPSS v19), with Time (first half of the task vs. second half of the task) and Emotion (positive vs. neutral) as the repeated factors, and PSWQ, MASQ-AA, and MASQ-AD-LI as continuous predictors. Of specific interest were the Time × Emotion × PSWQ and Time × Emotion × MASQ-AA interactions (depending on whether the ROI was associated with PSWQ or MASQ-AA). For brevity, only findings for these effects are reported. These analyses are only partially independent (Kriegeskorte et al. 2009), because the neutral condition was part of the contrast used to define the ROIs. However, the lack of complete independence biases toward finding patterns similar to those observed in the main analyses and thus actually biases against the test of specificity. Therefore, this bias renders the tests more conservative for present purposes.

#### Examination of incongruent stimuli

In order to ensure that present findings were driven by the negative valence of the stimuli, rather than by inhibition of distraction more generally, data from a color-word Stroop task (Stroop 1935) completed by the same set of participants were examined (for information on the task parameters, see Spielberg et al. 2011b). Data from four participants were excluded from these analyses because of poor data quality. Within-participant analyses were identical to those of the main analyses, with the exception that the task contrast of interest was incongruent minus congruent (as opposed to negative minus neutral). Higher-level analyses were identical to those used to examine specificity with regard to positive stimuli (i.e., both voxelwise and ROI analyses).

## Results

### Questionnaire descriptive statistics

PSWQ (*M* = 49.0, SD = 18.8) was correlated *r* = 0.53 (*P* < 0.001) with MASQ-AA (*M* = 26.8, SD = 7.2) and *r* = 0.53 (*P* < 0.001) with MASQ-AD-LI (*M* = 17.0, SD = 6.1), and MASQ-AA correlated *r* = 0.50 (*P* < 0.001) with MASQ-AD-LI. Means were within 1–2 points of both the means for the larger sample from which participants were drawn and published norms (Watson et al. 1995b; Startup and Erickson 2006).

### Behavioral analyses

A repeated-measures GLM for RT revealed an effect of Time (*F*_(1,74)_ = 17.2, *P* < 0.001), with RT increasing over time, and Emotion (*F*_(1,74)_ = 4.0, *P* = 0.049), with negative RT greater than neutral. The Emotion × Time interaction did not approach significance, and neither Time nor Emotion interacted with PSWQ, MASQ-AA, or MASQ-AD-LI. Similarly, the 3-way interactions between Time, Emotion, and psychopathology questionnaire were not significant, although the effects were in the predicted direction (PSWQ associated with increasing interference over time and MASQ-AA associated with decreasing interference over time).

A repeated-measures GLM for errors revealed an effect of Time (*F*_(1,74)_ = 5.0, *P* = 0.028), with errors increasing over time, and an effect of Emotion (*F*_(1,74)_ = 13.6, *P* < 0.001), with more errors in the negative condition. The Emotion × Time interaction was not significant, and neither Time nor Emotion interacted with PSWQ, MASQ-AA, or MASQ-AD-LI. Similarly, the 3-way interactions between Time, Emotion, and psychopathology questionnaire were not significant, although the effect was in the predicted direction for PSWQ (increasing error interference over time).

### fMRI analysis

Seven clusters exhibited a significant change across time in activation related to negative valence (see Table 3).

**Table 3:** Brain regions exhibiting a main effect of habituation

Region	Cluster size (mm^3^)	Mean *z*-value	Location		

			*X*	*Y*	*Z*
L Temporal Pole/pOFC/IFG/sACC (BA 11/25/38/47)	13,012	2.41	−24	23	−21
R Temporal Pole/STG (BA 38)	3463	2.44	45	15	−32
L STG/MTG/ITG (BA 20/21/22/37)	9785	2.36	−54	−32	−12
L Parahipp/Hipp/Amyg/Lingual Gyrus (BA 28/34/35/36/37)	4565	2.42	−22	−31	−17
R Lingual Gyrus/Occipital Pole (BA 17/18/19)	6502	2.37	11	−61	−5
R MTG/Lateral Occipital (BA 19/21/37)	6009	2.36	57	−57	4
B SFG/MFG/Frontal Pole (BA 6/8)	9319	2.31	0	31	50

L, left; R, right; B, bilateral; pOFC, posterior orbitofrontal cortex; IFG, inferior frontal gyrus; sACC, subgenual anterior cingulate; STG, superior temporal gyrus; MTG, middle temporal gyrus; ITG, inferior temporal gyrus; Parahipp, parahippocampal gyrus; Hipp, hippocampus; Amyg, amygdala; SFG, superior frontal gyrus; MFG, middle frontal gyrus; BA, Brodmann's area; Location, coordinates are for center of mass and are for ICBM152 2009a symmetrical space, with the *x*-axis moving from left to right.

#### Moderation of neural habituation

**Figure 1:**
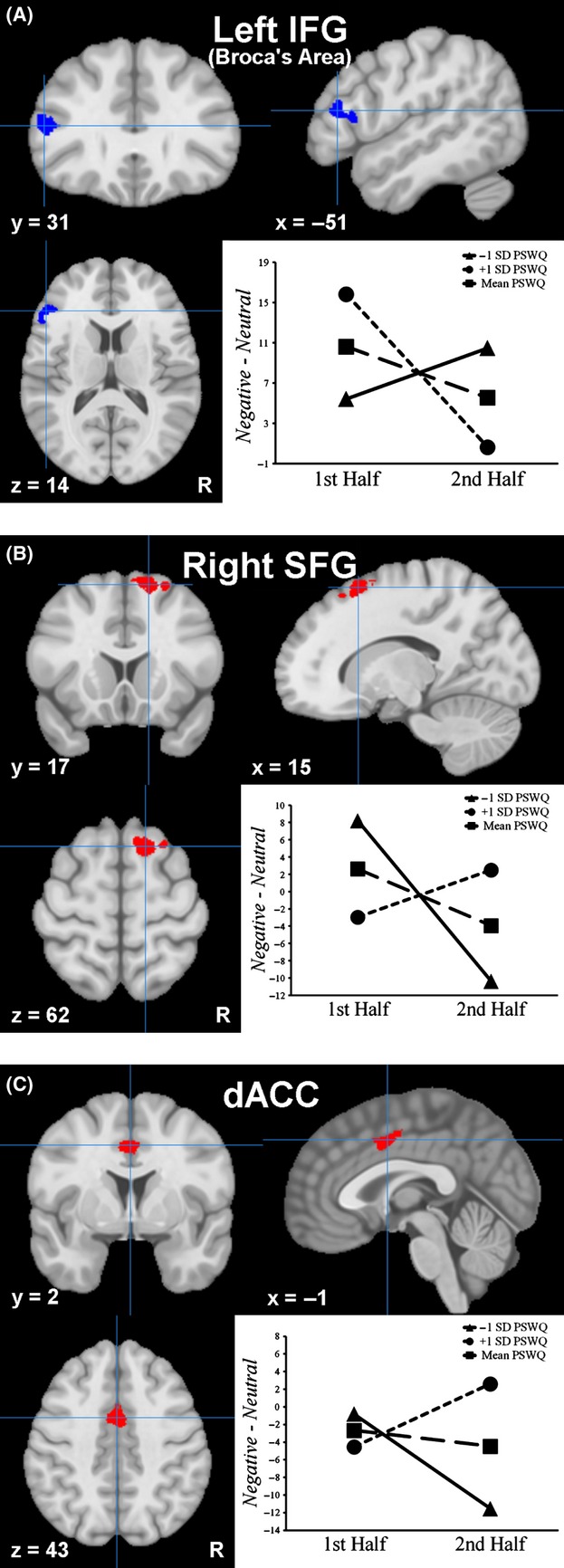
Moderation of habituation to negative stimuli by anxious apprehension. SFG, superior frontal gyrus; IFG, inferior frontal gyrus; dACC, dorsal anterior cingulate; Blue, high PSWQ associated with habituation; Red, high PSWQ associated with increased activation over time. The graphs depict the change in neural response to negative words over time at +1 (circle endpoints) and −1 (triangle endpoints) standard deviations (SD) and the mean (square endpoints) of the Penn State Worry Questionnaire (PSWQ). Time (1st task half and 2nd task half) is plotted on the *x*-axis against brain activation related to negative stimuli (negative minus neutral) on the *y*-axis. Graphs reflect values with normalized MASQ-AA and MASQ-AD-LI partialled out.

Table 4 lists brain regions in which change over time in activation to negative valence varied as a function of anxiety type. In line with hypotheses, PSWQ was associated with decreased activation (habituation) over time in Broca's area, shown in Figure 1A. As expected, PSWQ was associated with increased activation over time in two regions, right superior frontal gyrus (SFG; Fig. 1B) and dorsal ACC (dACC; Fig. 1C). The graphs in Figure 1 depict activation change in these clusters over time for +/− 1 standard deviations and the mean of PSWQ. Partial correlations between habituation of activation and anxious apprehension computed separately for negative and neutral words indicated that effects in Broca's area and right SFG were driven largely by changes in activation to negative stimuli, whereas the effect in dACC was driven largely by changes in activation to neutral stimuli (Table 5).

**Table 4:** Brain regions in which anxiety types moderated habituation

Region	Cluster size (mm^3^)	Direction of relationship	Mean *z*-value	Location		

				*X*	*Y*	*Z*
Anxious apprehension						
L IFG (Broca's area, BA 45/46)	1196	↓	2.30	−51	28	13
R SFG (BA 6/8)	1844	↑	−2.27	17	20	59
M dACC (BA 24)	1142	↑	−2.27	−1	1	43
Anxious arousal						
R MTG/ITG (BA 20/21)	2188	↓	2.28	64	−40	−10
M and R SFG (BA 6/8)	1616	↓	2.14	8	22	58
M Paracingulate (BA 6/8)	1478	↓	2.30	2	35	40
R MFG (DLPFC, BA 6/8/9)	5149	↓	2.25	44	22	32

L, left; R, right; M, medial; SFG, superior frontal gyrus; MFG, middle frontal gyrus; IFG, inferior frontal gyrus; DLPFC, dorsolateral prefrontal cortex; MTG, middle temporal gyrus; ITG, inferior temporal gyrus; dACC, dorsal anterior cingulate cortex; BA, Brodmann's area. ↑, Higher questionnaire scores associated with increased activation over time; ↓, Higher questionnaire scores associated with decreased activation over time; Location, coordinates are for center of mass and are for ICBM152 2009a symmetrical space, with the *x*-axis moving from left to right.

**Table 5:** Partial correlations between anxiety and habituation in activation for negative and neutral

Region	Habituation correlation for negative	*P*-value	Habituation correlation for neutral	*P*-value
Anxious apprehension				
L IFG (Broca's Area, BA 45/46)	−0.209	0.076	0.010	0.930
R SFG (BA 6/8)	0.215	0.068	0.003	0.983
M dACC (BA 24)	0.001	0.991	−0.188	0.112
Anxious arousal				
R MTG/ITG (BA 20/21)	−0.198	0.092	0.110	0.355
R SFG (BA 6/8)	−0.211	0.073	−0.012	0.919
M Paracingulate (BA 8/6)	−0.218	0.064	0.082	0.489
R MFG (DLPFC, BA 9/8/6)	−0.158	0.181	0.175	0.138

Pearson correlations are between habituation (2nd half vs. 1st half) in mean activation across each cluster and psychopathology questionnaire (PSWQ, MASQ-AA). Correlations were calculated separately for activation related to negative and neutral words. L, left; R, right; M, medial; SFG, superior frontal gyrus; MFG, middle frontal gyrus; IFG, inferior frontal gyrus; DLPFC, dorsolateral prefrontal cortex; MTG, middle temporal gyrus; ITG, inferior temporal gyrus; dACC, dorsal anterior cingulate cortex; BA, Brodmann's area.

**Figure 2:**
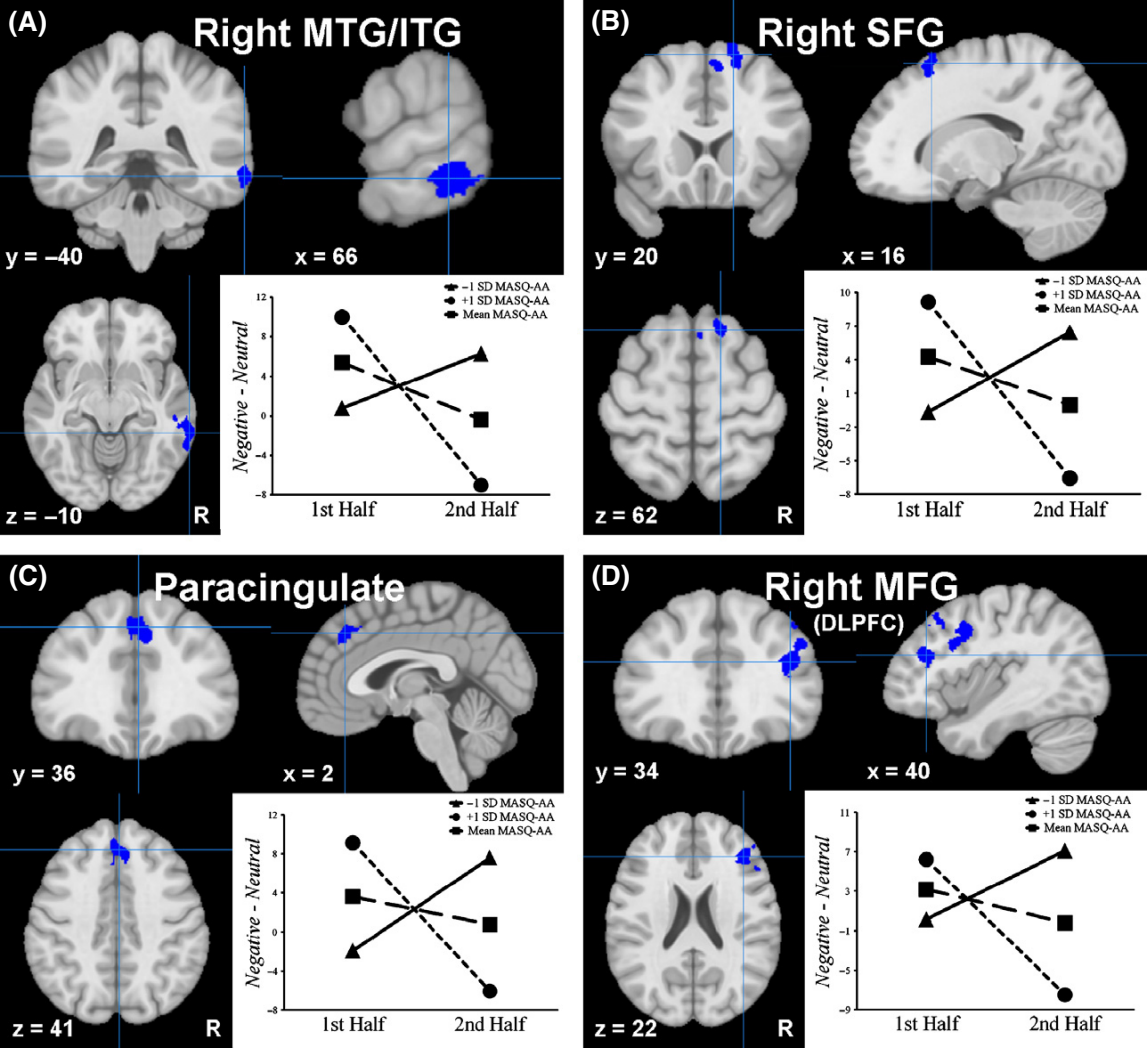
Moderation of habituation to negative stimuli by anxious arousal. SFG, superior frontal gyrus; MFG, middle frontal gyrus; DLPFC, dorsolateral prefrontal cortex; MTG, middle temporal gyrus; ITG, inferior temporal gyrus; Blue, high MASQ-AA associated with habituation. The graphs depict the change in neural response to negative words over time at +1 (circle endpoints) and −1 (triangle endpoints) standard deviations (SD) and the mean (square endpoints) of the Anxious Arousal subscale of the Mood and Anxiety Symptom Questionnaire (MASQ-AA). Time (1st task half and 2nd task half) is plotted on the *x*-axis against brain activation related to negative stimuli (negative minus neutral) on the *y*-axis. Graphs reflect values with normalized PSWQ and MASQ-AD-LI partialled out.

In line with hypotheses, MASQ-AA was associated with habituation in right MTG/ITG, shown in Figure 2A. As predicted, MASQ-AA was associated with habituation in three additional areas: right SFG (overlapping the right SFG area associated with PSWQ, despite analysis of unique variance), paracingulate, and right DLPFC (shown in Fig. 2B-D). The graphs in Figure 2 depict activation change in these clusters over time for +/− 1 standard deviations and the mean of MASQ-AA. As shown in Table 5, partial correlations indicated that all observed effects were driven largely by changes in activation to negative stimuli, except in right DLPFC, which appeared to be driven by changes in activation to both negative and neutral stimuli.

Given that PSWQ and MASQ-AA exhibited effects in opposite directions in right SFG, a direct test of these effects was computed. Two clusters were observed: one overlapping the right SFG regions observed for both PSWQ and MASQ-AA (center of mass = [14, 21, 59], cluster size = 1792 mm^3^, mean *z* = −2.36), and the second overlapping the right DLPFC cluster associated with MASQ-AA (center of mass = [43, 33, 27], cluster size = 1,448 mm^3^, mean *z* = −2.33). These findings indicate that the two anxiety types were associated with different responses to negatively valenced stimuli over time, with anxious arousal showing habituation and anxious apprehension showing either an increase (SFG) or no change (DLPFC) over time.

#### Psychophysiological interaction analyses

**Figure 3:**
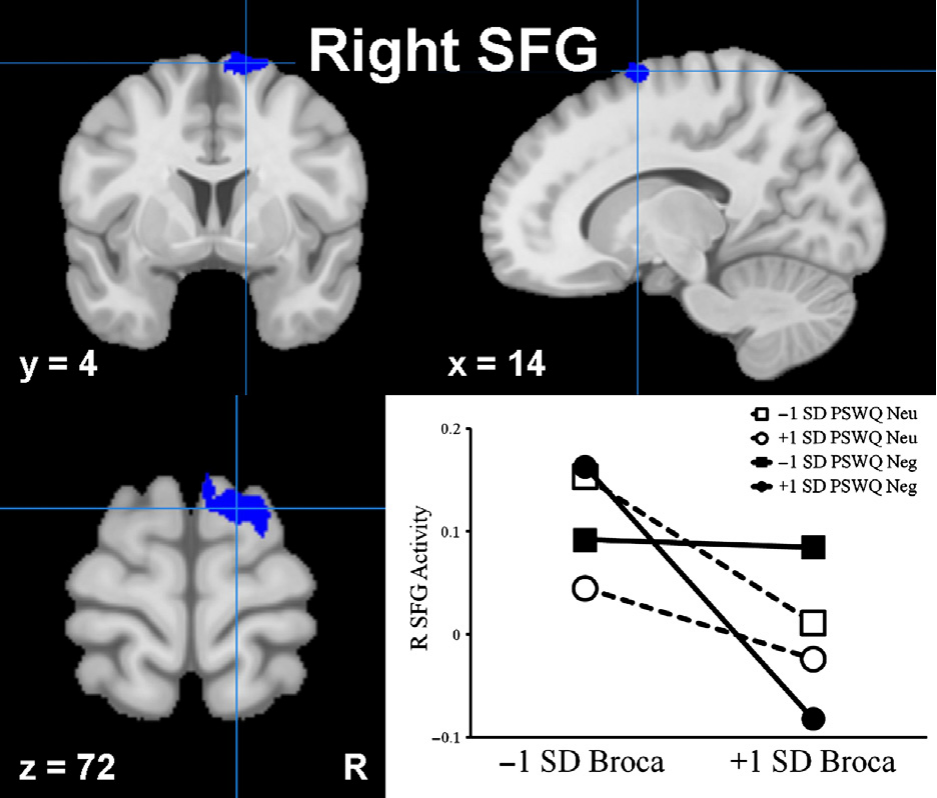
Moderation of connectivity between Broca's area and right superior frontal gyrus by anxious apprehension. SFG, superior frontal gyrus; Blue, high PSWQ associated with decreased connectivity with Broca's area; Neg, negative condition; Neu, neutral condition. The graph depicts connectivity between Broca's area and right superior frontal gyrus during the negative (solid lines) and neutral (dotted line) conditions at +1 (circle endpoints) and −1 (square endpoints) standard deviations (SD) of the Penn State Worry Questionnaire (PSWQ). Graph reflects values with (normalized) covariates partialled out.

As predicted, a cluster emerged in right SFG (listed in Table 6 and visualized in Fig. 3) in which PSWQ moderated condition-dependent changes in connectivity with Broca's area. This cluster was adjacent to, but did not overlap, the right SFG cluster associated with PSWQ identified in earlier analyses. As shown in Figure 3, the correlation between the two time series became more negative as PSWQ scores increased in the negative condition, with the relationship approaching 0 at low levels of PSWQ. Given that no relationships with PSWQ were observed in amygdala, and the effect in dACC was driven by changes to neutral rather than negative stimuli, only superior prefrontal cortex was examined for the PPI analyses.

**Table 6:** Region in which anxious apprehension moderated connectivity with Broca's area

Region	Cluster size (mm^3^)	Mean *z*-value	Location		

			*X*	*Y*	*Z*
R SFG (BA 6)	1286	−2.06	12	5	70

R, right; SFG, superior frontal gyrus; BA, Brodmann's area; Location, coordinates are for center of mass and are for ICBM152 2009a symmetrical space, with the *x*-axis moving from left to right.

The PPI remained significant when examined only in the first half of the task (*β* = −0.092, *P* = 0.006), whereas it was not significant when examined only in the second half of the task (*β* = −0.041, *P* = 0.229), although the effect was in the expected direction. No clusters emerged in which MASQ-AA or MASQ-AD-LI moderated connectivity with Broca's area.

#### Lateralization analyses

**Figure 4:**
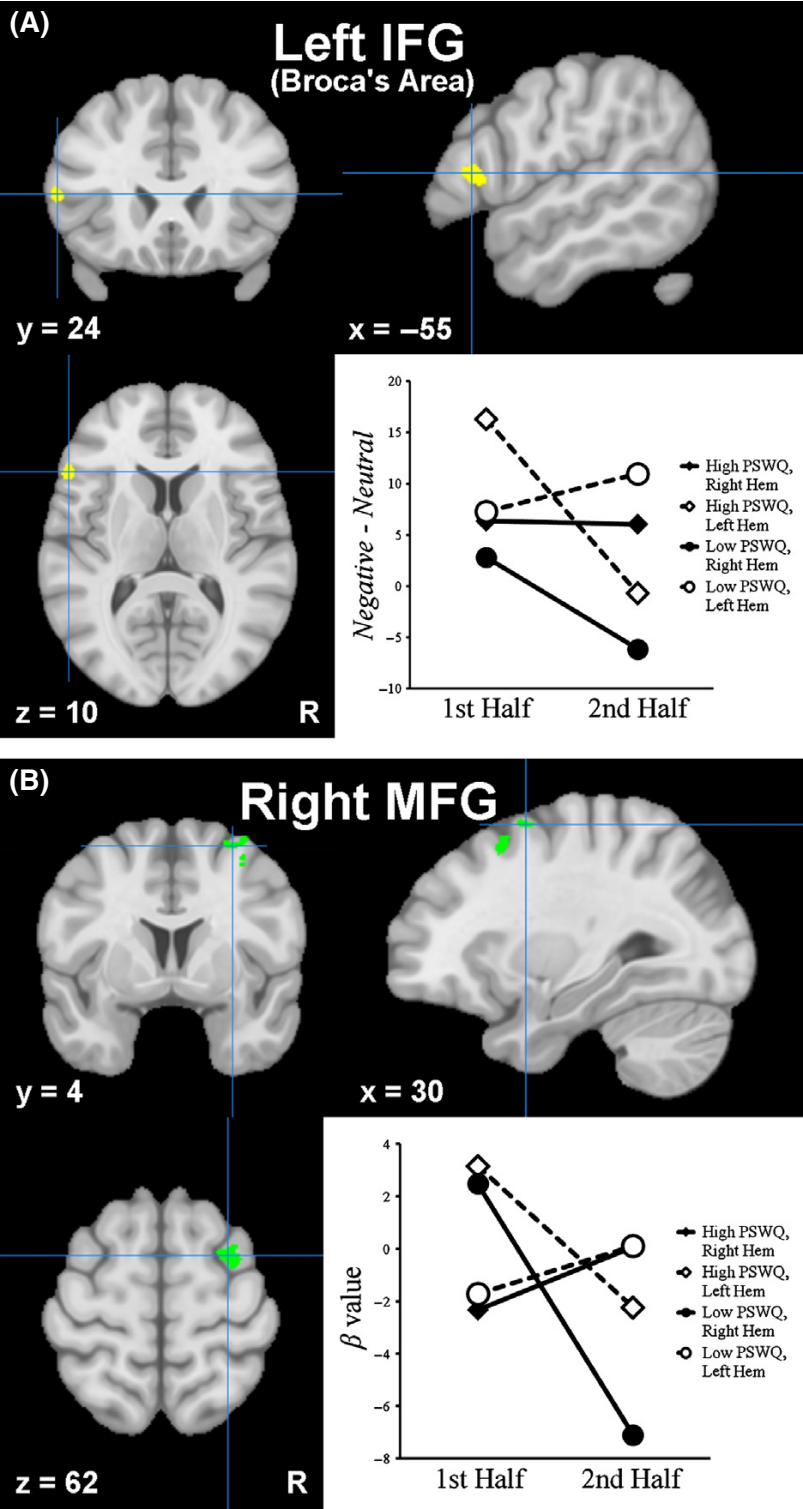
Lateralized moderation of habituation to negative stimuli by anxious apprehension. MFG, middle frontal gyrus; IFG, inferior frontal gyrus; Yellow, effect is left lateralized; Green, effect is right lateralized. The graphs depict the change in neural response to negative words over time in the left (dotted line) and right hemisphere (solid line) at +1 (diamond endpoints) and −1 (circle endpoints) standard deviations of the Penn State Worry Questionnaire (PSWQ). Time (1st task half and 2nd task half) is plotted on the *x*-axis against brain activation related to negative stimuli (negative minus neutral) on the *y*-axis. Graphs reflect values with normalized MASQ-AA and MASQ-AD-LI partialled out.

Consistent with hypothesis, PSWQ was associated with left lateralization in Broca's area.^8^ Although asymmetry was not found in SFG for PSWQ, right lateralization was observed in a nearby area of MFG (see Table 7 and Fig. 4).

**Table 7:** Brain regions in which moderation of habituation by anxiety types was lateralized

Region	Cluster size (mm^3^)	Direction of relationship	Mean *z*-value	Location		

				*X*	*Y*	*Z*
Anxious apprehension						
IFG^1^ (Broca's area, BA 45)	274	L > R	2.79	−55	22	9
MFG (BA 6)	1165	R > L	2.01	34	11	54
Anxious arousal						
MTG/ITG^2^ (BA 20/21)	554	R > L	2.11	66	−43	−11
MFG (BA 6)	2078	R > L	2.23	33	0	62
MFG (DLPFC, BA 9)	1544	R > L	2.09	43	30	28

L, left; R, right; MFG, middle frontal gyrus; IFG, inferior frontal gyrus; DLPFC, dorsolateral prefrontal cortex; MTG, middle temporal gyrus; ITG, inferior temporal gyrus; BA, Brodmann's area; Location, coordinates are for center of mass and are for ICBM152 2009a symmetrical space, with the *x*-axis moving from left to right.

1 individual voxel threshold of *P* = 0.04.

2 individual voxel threshold of *P* = 0.02.

**Figure 5:**
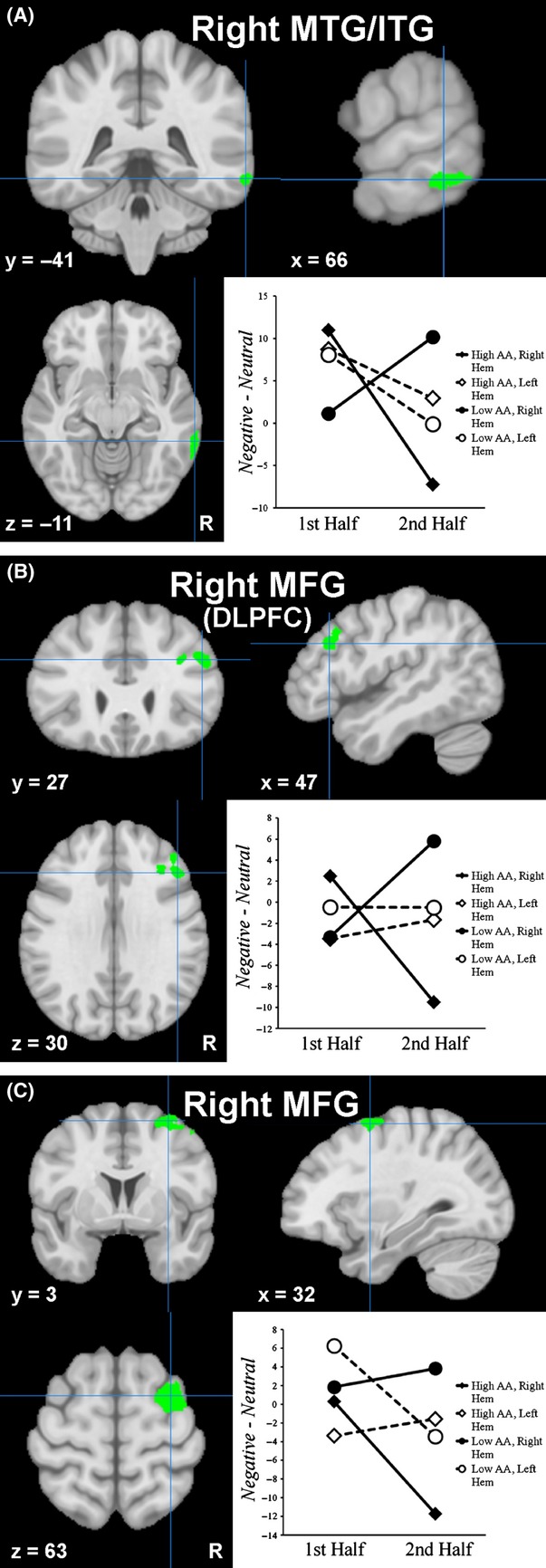
Lateralized moderation of habituation to negative stimuli by anxious arousal. MFG, middle frontal gyrus; DLPFC, dorsolateral prefrontal cortex; MTG, middle temporal gyrus; ITG, inferior temporal gyrus; Green, effect is right lateralized. The graphs depict the change in neural response to negative words over time in the left (dotted line) and right (solid line) hemisphere at +1 (diamond endpoints) and −1 (circle endpoints) standard deviations of the Anxious Arousal subscale of the Mood and Anxiety Symptom Questionnaire (AA). Time (1st task half and 2nd task half) is plotted on the *x*-axis against brain activation related to negative stimuli (negative minus neutral) on the *y*-axis. Graphs reflect values with normalized PSWQ and MASQ-AD-LI partialled out.

In line with hypotheses, MASQ-AA was associated with right lateralization in MTG/ITG^9^ and DLPFC. Additionally, although asymmetry was not found in SFG for MASQ-AA, right lateralization was observed in a nearby area of MFG (see Table 7 and Fig. 5).

#### Anxious apprehension ROI mediation analyses

Activation changes in Broca's area and right SFG ROIs identified in the main analyses were tested as mediators of the effect of anxious apprehension on behavior (dACC was not examined, because the effect was driven by changes to neutral rather than negative stimuli). Results indicated that activation in Broca's area mediated the effect of PSWQ on both RT and errors, whereas activation in right SFG did not (although the effect was in the expected direction and approached significance for RT). Findings support the hypothesis that anxious apprehension is associated with changes in behavior over time but that these effects are masked in the zero-order relationship between anxious apprehension and behavior.

Specifically, both of the paths from PSWQ to the mediators were significant (Broca's area: *β* = −0.52, *P* = 0.003; SFG: *β* = 0.61, *P* = 0.001), as expected. The paths between Broca's area and both RT and error habituation were significant (RT: *β* = 1.52, *P* < 0.001; errors: *β* = 0.05, *P* = 0.047), whereas the paths between right SFG and RT/errors were not (RT: *β* = 0.45, *P* = 0.154; errors: *β* = −0.00, *P* = 0.849). Finally, the indirect effects of PSWQ on RT and errors through Broca's area were significant (RT: *β* = −0.79, bias-corrected 95% confidence interval = [−1.54, −0.25]; errors: *β* = −0.03, confidence interval = [−0.07, −0.00]), whereas the indirect effect through right SFG only approached significance for RT (*β* = 0.28, confidence interval = [−0.01, 0.69]) and was not significant for errors (*β* = −0.00, confidence interval = [−0.04, 0.03]).

### Tests of specificity to negative stimuli

#### Examination of positive stimuli

In the voxelwise analyses, no clusters were observed in which PSWQ or MASQ-AA moderated habituation to positive stimuli, and the ROIs from the main analyses did not exhibit significant habituation to positively valenced stimuli,^10^ supporting the hypothesis that present findings were specific to negative stimuli.

#### Examination of incongruent stimuli

In the voxelwise analyses, no clusters were observed in which PSWQ moderated habituation to incongruent stimuli. Additionally, the ROIs from the main analyses did not exhibit significant habituation to incongruent stimuli,^11^ supporting the hypothesis that present findings are specific to negative stimuli.

One cluster was observed in right MFG in which MASQ-AA moderated habituation (center of mass = [36, 7, 56], cluster size = 2,414 mm^3^, mean *z* = −2.23), and this cluster negligibly overlapped the right DLPFC cluster associated with MASQ-AA (28 mm^3^, <1% of DLPFC cluster). In contrast to the findings from the main analyses, MASQ-AA was associated with increased activation to incongruent stimuli over time in this cluster, further confirmation that present findings were specific to negative stimuli.

## Discussion

As hypothesized, anxious arousal was associated with habituation to negative stimuli in right MTG/ITG and three other areas: right DLPFC, right SFG, and medial paracingulate. Also as predicted, anxious apprehension was associated with habituation in Broca's area and a concurrent increase in activation over time in right SFG. Anxious apprehension was also associated with activation change over time in medial dACC, although this finding appeared to be driven by decreased activation over time to neutral stimuli rather than an increase to negative stimuli. Importantly, the two anxiety types exhibited opposing patterns of activation change in right SFG, with anxious apprehension associated with increased activation over time and anxious arousal with habituation. In addition, present findings appear to be specific to negatively valenced stimuli (as opposed to positively valenced or distracting, neutrally valenced stimuli). Overall, present findings support the hypothesis that an immediate response to negatively valenced stimuli is enhanced in individuals with elevated levels of anxious arousal but is delayed in individuals with elevated levels of anxious apprehension. Thus, the two anxiety types appear to be characterized by differences in a neural manifestation of affective time course; specifically, anxious arousal exhibited a faster rise time to full engagement with negatively valenced words, along with a more rapid recovery to baseline.

### Habituation associated with anxious arousal

Present neural findings support assertions that anxious arousal is associated with engagement of a threat surveillance system (Nitschke et al. 2000). Habituation was observed in several areas that are part of a model of the neural instantiation of attention proposed by Corbetta et al. (2008). Specifically, habituation was observed in right MTG/ITG, which has consistently been associated with bottom-up, stimulus-driven attention, and right DLPFC, which has consistently been associated with top-down biasing of attention, along with stimulus-driven interruption of attention (Corbetta et al. 2008). Additionally, the SFG cluster observed in the present study may overlap with FEF, although FEF is often located posterior to this at the intersection of the superior frontal and superior precentral sulci (e.g., Kincade et al. 2005; Curtis and D'Esposito 2006). However, the MFG cluster exhibiting a lateralized effect (adjacent to the SFG cluster) is located in the area typically labeled FEF, which has also been associated with top-down biasing of attention (Corbetta et al. 2008). Overall, present findings support the hypothesis that anxious arousal is associated with habituation in attention to negative stimuli, although this effect was not observed in overt behavior.

Although these attention-related regions are thought to be activated in relation to any type of goal, there is evidence of hyperactivation in these regions when threat is encountered. Specifically, the clusters associated with anxious arousal in the present study are hyperactive when participants view threat-related stimuli (Ashwin et al. 2007) or are threatened with unpredictable painful physical stimulation (Carlsson et al. 2006). Additionally, hyperactivation has been observed in these areas when individuals with anxiety disorders encounter disorder-relevant stimuli (e.g., spider pictures for individuals with spider phobia, Goossens et al. 2007). Finally, these areas are activated by ambiguity during decision-making tasks (Volz et al. 2005). Interestingly, this response appears to be specific to ambiguity rather than risk (where the probabilities of winning and losing are known, but the outcome is not, Krain et al. 2006), suggesting that these areas are activated by a need for more information rather than the mere possibility of danger (see Shackman et al. 2009). In summary, these areas appear to be sensitive to unexpected cues signaling potential threat.

In addition to areas overlapping with the attentional network proposed by Corbetta et al. (2008), anxious arousal was also associated with habituation in paracingulate. This area responds when participants are threatened with painful physical stimulation (Jensen et al. 2003) or when presented with uncertainty during decision-making (Volz et al. 2005). Additionally, this area has exhibited hyperactivation when individuals with obsessive–compulsive disorder encounter stimuli related to compulsive checking (stimuli that engender uncertainty, Mataix-Cols et al. 2004). This research is consistent with a recent proposal that this region, along with nearby cingulate, is involved in adapting behavior in uncertain situations based on information gained from aversive outcomes (Shackman et al. 2011b).

Present findings are consistent with a proposed threat monitoring system that includes the right MTG/ITG area and right MFG (Nitschke et al. 2000). This system is hypothesized to monitor for, and reorient toward, potential threat and to exert top-down control when threat is detected in order to respond effectively. Evidence suggests that hyperactivation of this system is associated with the attentional biases found in anxiety (Nitschke et al. 2000). Taken together with present findings, the research reviewed above indicates that anxious arousal is associated with immediate activation of a threat surveillance system, and that this activation diminishes over time. This suggests that anxious arousal is associated with initial identification of negative stimuli as salient and potentially threatening but that this perception weakens over time as stimuli become more familiar and predictable.

Enhanced monitoring for, and reactivity to, negatively valenced information is adaptive in some situations. However, it may also lead to a chronic increase in distress in individuals with high levels of anxious arousal, because these individuals consistently overidentify cues predictive of threat. In turn, this may foster irrational fears (e.g., specific phobias) and/or panic attacks, because the likelihood of encountering threats is overestimated. However, the association between anxious arousal and habituation in attention-related brain regions indicates that individuals high in anxious arousal will be particularly amenable to exposure-based interventions, because habituation during exposure is predictive of recovery from anxiety disorders (Jaycox et al. 1998).

### Habituation associated with anxious apprehension

Results revealed habituation in the response to negatively valenced stimuli in Broca's area. Given the consistent association between Broca's area and verbal rehearsal (Zatorre et al. 1996), of which worry is a subset (Borkovec and Inz 1990), along with evidence of hyperactivation of Broca's area in individuals who consistently engage in worry (Engels et al. 2007), a reasonable interpretation of this habituation is that it reflects decreased engagement in worry over time (although see the Limitations section below). A decrease in worry over time is consistent with evidence that worry is cognitively taxing and engages resources that can be depleted with continued use (Hayes et al. 2008). As activation in Broca's area decreased over time, response to negative words increased in right SFG (and a lateralized effect appeared in right MFG). As discussed above, these areas are in or adjacent to FEF, which has been associated with top-down biasing of attention. Therefore, a potential interpretation is that the findings in right SFG/MFG indicate that anxious apprehension is associated with increased attention to negative stimuli over time.

Although these findings are consistent with the hypothesis that habituation in Broca's area is associated with a concurrent increase in activation in attention-related areas, they do not represent a direct test of this hypothesis. Therefore, direct tests were conducted using PPI analyses, which indicated that Broca's area time series was negatively correlated with the time series of a right SFG cluster (adjacent to the SFG cluster identified in earlier analyses) during the negative word condition, and the magnitude of this relationship was larger in individuals high in anxious apprehension. This finding is important, because it provides more direct support for the hypothesis that the opposing pattern of activation change over time in these areas is due to the influence (direct or indirect) of Broca's area on right SFG. Given that the present analyses do not assess causality or direction of effect, this inference is very preliminary. Rather, the present finding serves to support the existence of a relationship between these regions, and future research should assess its direction and causality.

No direct relationship was found between anxious apprehension and behavior. However, present findings partially supported the hypothesis that the effect of anxious apprehension on habituation of behavior is mediated, in opposing directions, by Broca's area and right SFG. Specifically, there was a significant indirect effect through Broca's area, with anxious apprehension positively associated with habituation, whereas the indirect effect through SFG was not significant, although in the hypothesized direction for RT (i.e., anxious apprehension negatively associated with habituation). Therefore, present findings suggest that anxious apprehension is associated with habituation in behavior, although additional mediators likely remain to be discovered.

The findings that anxious apprehension was associated with decreased response in dACC over time to neutral words and no change over time in response to negative words was unexpected. Research suggests that this dACC area is involved in the selection of appropriate actions (Picard and Strick 2001) and is part of a network of areas that support initiation and maintenance of task sets (Dosenbach et al. 2008). Therefore, it is possible that participants high in anxious apprehension are initially concerned about performing tasks adequately and that they engage dACC in order to appropriately select actions. The fact that this happens only when the stimuli are not negatively valenced may reflect the fact that worry evoked by negative words supersedes the desire to perform the task adequately.

Overall, the anxious apprehension findings provide further support for Borkovec's theory that worry functions as a form of cognitive avoidance (Borkovec et al. 2004), because initial engagement of Broca's area in individuals high in anxious apprehension was associated with a suppressed initial response in right SFG. If engagement with negative stimuli is suppressed by worry, the aversive experience of fully processing negatively valenced stimuli may be avoided, which may reinforce engagement in worry. Present findings also support the hypothesis that engaging in cognitive avoidance can disrupt habituation, because activation in right SFG increased over time rather than habituated. Thus, although engaging in worry may be an adaptive response to negatively valenced stimuli in the short term, the associated disruption in habituation may lead to the maintenance of anxiety over time. Consequently, engagement in worry may disrupt exposure-based interventions, and worry reduction before engaging in exposure may increase the effectiveness of such interventions. In addition, findings may provide insight into the mechanisms of effectiveness of mindfulness-based treatments for anxiety (Orsillo et al. 2005). To the degree that the practice of mindfulness disrupts worry via a focus on attention to breathing and not on thoughts, natural or therapeutic exposure to feared stimuli will produce habituation more successfully. Thus, mindfulness-based interventions may have a greater therapeutic impact on individuals with excessive anxious apprehension than on those whose primary difficulty is excessive anxious arousal.

Although the present study focused on worry, the findings for anxious apprehension may be due specifically to the repetitive nature of worry. If so, findings may also apply to other patterns of repetitive thinking such as rumination and may be better captured by a higher-order factor sometimes labeled repetitive negative thinking (McEvoy et al. 2010) or perseverative iterative thinking style (Davey and Levy 1998). Given that repetitive thinking patterns are common to a number of psychopathology dimensions (e.g., depression), present findings may indicate that reducing repetitive thinking is an important first step in treatment more generally. Alternatively, if present findings are due to the unique features of worry, which remain a matter of debate (Papageorgiou 2006), a reduction in repetitive thinking may be useful only in the treatment of worry.

### Strengths and limitations

The present study benefited from a large sample relative to the fMRI literature. It extends the literature by simultaneously examining anxious apprehension and anxious arousal, as opposed to examining only one anxiety type or ignoring the distinction. Given that these anxiety types are correlated, inferences based on the measurement of only one anxiety type may not reflect effects specific to that anxiety type. The study also benefited from statistically controlling comorbid depression, which often confounds studies of anxiety due to shared general distress. Additionally, the present study examined habituation at the level of neural activation, which can reveal effects that may cancel out at the level of behavior.

However, present findings should be interpreted in the context of some limitations. First, although the present study purposefully chose stimuli that would not elicit an extremely strong fear response, in order to foster habituation, the stimuli may not be as relevant to pathological anxiety as stimuli that evoke a much stronger fear response (e.g., spiders for individuals with spider phobia). Second, it is unclear whether the stimuli used in the present investigation were experienced as threatening, and this may have limited the level of fear experienced by participants. Future research could explore a variety of stimuli that may engage fear more strongly.

Third, the present study interprets the observed habituation in Broca's area as reflecting habituation in worry. However, it is possible that the activation in Broca's area observed in the present study reflects processes other than, or in addition to, worry. For example, it is possible that Broca's area activation better reflects engagement in processing of the specific word stimuli, rather than actual verbal rehearsal of worries. Even if true, engagement in word processing may still be driven by worry (e.g., worry about performing the task or word meaning), an inference that seems reasonable given that anxious apprehension moderated this activation. One possible method of differentiating between worry and word processing specific to the stimuli would be to disentangle block-level and stimulus-specific variance, given that worry should be increased during negative blocks but not necessarily tied to specific stimuli. Although there is jitter in the presentation of words in the present study, the length of the jitter is small compared to the HRF, and the timing of the stimuli (2000 ± 225 msec) is approximately equal to the fMRI sampling rate (TR = 2 sec). In order to differentiate between these signals without aliasing, TR would have to be less than 1 sec or ITI greater than 4 sec. Therefore, the present design does not allow for an appropriate differentiation between these sources of variance, and caution must be taken in making inferences about the psychological processes instantiated in Broca's area.

Finally, the present study found changes over time in brain activation, but these changes were not reflected in significant direct behavioral effects. An indirect effect of anxious apprehension on behavior through Broca's area was observed, along with a nonsignificant effect through right SFG that was in the expected direction for RT. Therefore, it is possible that more power is needed to detect the SFG indirect effect and/or there may be as yet unidentified regions that mediate effects that are in the direction opposite to that of Broca's area. Additionally, given that the hypothesized behavioral effect for anxious arousal was in the expected direction, although far from significant, lack of significance here may also be due to a lack of power rather than a true absence of behavioral findings. Furthermore, researchers have begun to caution against discounting neural findings because behavioral effects are not evident, given that behavior is not necessarily the gold standard for the occurrence of relevant psychological processes (e.g., Wilkinson and Halligan 2004). However, caution should be maintained when making inferences about present findings given the lack of significant direct behavioral effects. Future research with either more potent threat stimuli (which may have a larger effect on behavior) or a sample size large enough to detect small overt behavioral effects is needed in order to determine whether the patterns of behavioral habituation associated with anxious arousal and apprehension are consistent with the present neural findings.

In summary, the present study provides evidence for differential time courses of neural habituation associated with anxious apprehension and anxious arousal in line with Corbetta et al.'s (2008) theory of brain mechanisms serving attention and Heller and colleagues (Heller et al. 1995, 1997; Nitschke et al. 1999, 2000; Engels et al. 2007, 2010) distinction between these two types of anxiety. Present findings suggest that anxious arousal is associated with immediate engagement with negative stimuli and a concurrent recruitment of an attentional surveillance system, followed by habituation of this response. In contrast, anxious apprehension is associated with immediate engagement in worry and delayed engagement with negatively valenced stimuli that occurs only after worry decreases.

## Footnotes

1 The present sample partially overlapped the samples used in Engels et al. (2010) and Spielberg et al. (2012) by 89% and 92%, respectively. However, these studies did not examine variance in activation across time or connectivity between brain regions. Therefore, the studies report independent findings.

2 In order to ensure that the use of only the 8-item subscale did not bias the findings, analyses were also conducted with the full 22 item Anhedonic Depression scale, with virtually identical results.

3 It is possible that between-session habituation occurred for those participants who had already participated in the task during the EEG session, which could have led to different patterns of habituation for these participants, relative to those participants who had not already participated in the task. We tested this possibility by examining whether moderation of neural habituation by anxiety type differed for those who had already participated in the EEG task and those who had not. Results revealed that there was no significant difference in moderation of habituation by anxiety type. This finding is consistent with research indicating that, even when within-session habituation occurs, multiple sessions of exposure may occur before between-session habituation is evident, and peak fear activation may actually increase between early sessions (e.g., Nishith et al. 2002).

4 In order to assess whether the specific stimuli used in the present study can elicit fear, we examined ratings of the ANEW data set provided by Stevenson et al. (2007). In this study, participants rated whether each word elicited fear (rating scale: [1] “not at all” to [5] “extremely”). We extracted the mean fear rating (across participants) for each of the words used in the present study and conducted *t*-tests to determine (1) whether words from the negative condition elicited significant levels of fear and (2) whether words from the negative condition elicited significantly greater fear than did words from the neutral condition. First, a one-sample *t*-test indicated that words from the negative condition elicited significant levels of fear (mean = 3.3, *t*_(63)_ = 31.4, *P* < 0.001). Second, an independent samples *t*-test indicated that words from the negative condition elicited significantly greater levels of fear than did words from the neutral condition (mean difference = 1.9, *t*_(190)_ = 29.8, *P* < 0.001). Therefore, the negative words used in the present study can elicit fear.

5 The relationship between MASQ-AD-LI and neural habituation was examined using a whole-brain gray-matter mask, because no a priori hypotheses were made regarding depression and habituation in specific brain areas. No significant clusters were observed in which MASQ-AD-LI moderated habituation to negative stimuli.

6 The Broca's area mask from the Juelich atlas (standard with FSL) was also examined. All clusters found using the IFG mask were also observed when using the Juelich Broca's area mask, indicating that choice of mask did not drive present findings.

7 It was possible that the PSWQ analyses were biased to be more liberal that the MASQ-AA analyses, because two smaller masks were used for the PSWQ analyses rather than one large mask (two masks were used because the tests for left IFG vs. the other regions were one-tailed in opposite directions). In order to ensure that this did not bias the findings, cluster thresholds were computed for a mask that combined all the regions tested for PSWQ (Broca's area, superior prefrontal cortex, ACC, and amygdala). Results for PSWQ remained significant at this threshold, indicating that analyses were not biased.

8 This cluster was present only at a higher individual voxel-level threshold of *P* = 0.02, still corrected for multiple comparisons.

9 The fact that the MTG/ITG cluster in which MASQ-AA moderated habituation was located largely in the superior–posterior quadrant of the MTG/ITG mask rendered testing of a large portion of that mask unnecessary. Therefore, everything anterior and inferior to the midpoint of that mask (*y* = −31, *z* = −15) was zeroed. The midpoint was used to divide the mask (as opposed to boundaries of the observed cluster) so as not to bias tests of laterality.

10 For the ROI analyses of the Broca's area and right SFG clusters associated with PSWQ, the Time × Emotion × PSWQ interactions were not significant (Broca's area: *F*_(1,71)_ = 0.0, *P* = 0.99; SFG: *F*_(1,71)_ = 0.2, *P* = 0.67; dACC: *F*_(1,71)_ = 1.7, *P* = 0.20). The Time × Emotion × MASQ-AA interactions for MASQ-AA ROIs were not significant (MTG/ITG: *F*_(1,71)_ = 2.1, *P* = 0.15; SFG: *F*_(1,71)_ = 0.8, *P* = 0.38; paracingulate: *F*_(1,71)_ = 2.4, *P* = 0.13), with the exception of right DLPFC (*F*_(1,71)_ = 4.2, *P* = 0.05). In order to determine whether the interaction in DLPFC was driven by changes in the positive as well as the neutral condition, partial correlations between MASQ-AA and habituation in brain activation were computed separately for the two conditions. The partial correlations for positive and neutral conditions were −0.04 (*P* = 0.75) and 0.18 (*P* = 0.14), respectively, indicating that this finding was driven by changes in the neutral rather than the positive condition. In summary, ROI analyses indicate that present findings are specific to negative stimuli for all clusters.

11 For the ROI analyses, neither the Time × Emotion × PSWQ interactions (Broca's area: *F*_(1,67)_ = 0.0, *P* = 0.84; SFG: *F*_(1,67)_ = 0.3, *P* = 0.61; dACC: *F*_(1,67)_ = 0.0, *P* = 0.94) nor the Time × Emotion × MASQ-AA interactions (MTG/ITG: *F*_(1,67)_ = 2.2, *P* = 0.14; DLPFC: *F*_(1,67)_ = 2.3, *P* = 0.13; SFG: *F*_(1,67)_ = 1.4, *P* = 0.25; paracingulate: *F*_(1,67)_ = 0.0, *P* = 0.96) were significant.
